# State-Specific Prevalence of Depression Among Adults With and Without Diabetes — United States, 2011–2019

**DOI:** 10.5888/pcd20.220407

**Published:** 2023-08-10

**Authors:** Alain K. Koyama, Israel A. Hora, Kai McKeever Bullard, Stephen R. Benoit, Shichao Tang, Pyone Cho

**Affiliations:** 1 Division of Diabetes Translation, National Center for Chronic Disease Prevention and Health Promotion, Centers for Disease Control and Prevention, Atlanta, Georgia

## Abstract

**Introduction:**

In 2019 among US adults, 1 in 9 had diagnosed diabetes and 1 in 5 had diagnosed depression. Since these conditions frequently coexist, compounding their health and economic burden, we examined state-specific trends in depression prevalence among US adults with and without diagnosed diabetes.

**Methods:**

We used data from the 2011 through 2019 Behavioral Risk Factor Surveillance System to evaluate self-reported diabetes and depression prevalence. Joinpoint regression estimated state-level trends in depression prevalence by diabetes status.

**Results:**

In 2019, the overall prevalence of depression in US adults with and without diabetes was 29.2% (95% CI, 27.8%–30.6%) and 17.9% (95% CI, 17.6%–18.1%), respectively. From 2011 to 2019, the depression prevalence was relatively stable for adults with diabetes (28.6% versus 29.2%) but increased for those without diabetes from 15.5% to 17.9% (average annual percent change [APC] over the 9-year period = 1.6%, *P* = .015). The prevalence of depression was consistently more than 10 percentage points higher among adults with diabetes than those without diabetes. The APC showed a significant increase in some states (Illinois: 5.9%, Kansas: 3.5%) and a significant decrease in others (Arizona: −5.1%, Florida: −4.0%, Colorado: −3.4%, Washington: −0.9%). In 2019, although it varied by state, the depression prevalence among adults with diabetes was highest in states with a higher diabetes burden such as Kentucky (47.9%), West Virginia (47.0%), and Maine (41.5%).

**Conclusion:**

US adults with diabetes are more likely to report prevalent depression compared with adults without diabetes. These findings highlight the importance of screening and monitoring for depression as a potential complication among adults with diabetes.

**Summary**

**What is already known about this topic?**

Depression and diabetes have a bidirectional relationship and are both highly prevalent conditions that frequently occur together among adults in the US.

**What is added by this report?**

Compared with those without diabetes, US adults with diabetes consistently had a higher prevalence of depression during 2011 through 2019. Trends in prevalence of depression in adults with diabetes were stable in most states, though significantly increased or decreased in other states.

**What are the implications for public health practice?**

Our findings highlight both the substantial burden of depression in adults with diabetes and the need for effective prevention and management for both conditions.

## Introduction

Though diabetes incidence has begun to decline, prevalence remains high, and therefore the burden of diabetes complications persists as a major public health concern (1). Much of the research and clinical focus on diabetes complications involves conditions such as cardiovascular disease, kidney disease, and neuropathy rather than mental health. While end-organ damage can substantially contribute to a lower quality of life and increased deaths, mental health conditions such as depression can negatively affect management of diet, adequate physical activity, smoking cessation, glycemic control, and medication adherence (2). Therefore, whether indirectly through poor disease management or through potentially direct biologic mechanisms (3), prevention of depression can be vital to improve diabetes outcomes.

In 2019 in the US, about 1 in 9 adults had diagnosed diabetes (1), and 1 in 5 adults had diagnosed depression (4). While the prevalence of diabetes increases with age, depression tends to be more prevalent among younger age groups (5). Disparate trends are also observed by sex, where men are more likely to have diabetes than women, but women are more likely to have depression than men (4). Both conditions are more prevalent among adults of low socioeconomic status (6,7). Among adults with diabetes, measuring prevalence of depression can be essential to evaluating its potential to affect diabetes outcomes and to highlight any sociodemographic or geographic disparities in prevalence.

Although the national prevalence of mental health conditions, including depression, among adults with diabetes has been reported (8,9), to our knowledge information on state-specific prevalence estimates of depression among adults with diabetes is limited. Additionally, previous studies were published before diabetes incidence began to decline in the US, therefore an updated analysis is merited because the prevalence of comorbid conditions may have also changed. We assessed the state-specific prevalence of diagnosed depression among adults in the US from 2011 through 2019, evaluating trends over time as well as prevalence within sociodemographic subgroups.

## Methods

### Study design and setting

We conducted a secondary data analysis of the Behavioral Risk Factor Surveillance System (BRFSS), an annual state-based landline and cellular telephone survey of a randomly selected representative sample of noninstitutionalized adults aged 18 years or older, in all 50 states of the US, the District of Columbia, and participating US territories and Affiliated Pacific Islands. The BRFSS survey is administered and supported by the National Center for Chronic Disease Prevention and Health Promotion, at the Centers for Disease Control and Prevention (CDC). Health departments in each state follow guidelines provided by CDC to conduct the interviews and then transmit data to CDC for editing, processing, and weighting to create a nationally representative data set. Deidentified line-level data are then made publicly available for analysis. For the landline telephone survey, interviewers collect data from a randomly selected adult in a household. For the cellular telephone survey, interviewers collect data from adults answering the telephone who reside in a private residence or college housing. The survey collects information from survey respondents on health-related behavioral risk factors, health care access, and chronic conditions. Major changes to the BRFSS survey methods occurred in 2011, and the assessment on status of diabetes and depression has appeared on the chronic health conditions module as annual core questions since then. Thus, we selected our 9-year study period from 2011 through 2019. For each year during the study period, BRFSS sample sizes ranged between 418,268 (in 2019) and 506,467 (in 2011). The overall median survey response rate also varied from 45.2% (2012) to 49.9% (2018). From 2011 through 2019, 4,102,152 respondents were interviewed in all 50 states and the District of Columbia. A total of 26,082 were excluded for missing data on depression or diabetes status or both, resulting in an analytic sample of 4,076,070 total respondents. Additional information on BRFSS methodology can be found elsewhere (4). Analyses of BRFSS data are deemed exempt from review by CDC’s institutional review board.

### Measurements

Data were based on self-reported information. Demographic information included age, sex (male, female), and race and ethnicity (Hispanic, non-Hispanic Black, non-Hispanic White, non-Hispanic other [includes participants who identified as American Indian and Alaska Native, Non-Hispanic Asian and Pacific Islander, or other]). Socioeconomic variables included educational attainment (less than high school graduate, high school graduate or equivalent, some college, college graduate), annual household income (<$35,000; $35,000–<$50,000; $50,000–<$75,000; ≥$75,000), and health insurance coverage (insured, uninsured). Diagnosed diabetes status was determined by the participant’s yes response to the question, “Has a doctor, nurse, or other health professional ever told you that you had diabetes?” To exclude gestational diabetes, female respondents were asked if they had been told they had diabetes other than during pregnancy. Diagnosed depression was also defined as a yes response to the question “Has a doctor, nurse, or other health professional ever told you that you had a depressive disorder (including depression, major depression, dysthymia, or minor depression)?”

### Statistical analysis

First, we examined sociodemographic characteristics of US adults aged 18 years or older by self-reported diabetes status in all 50 states and the District of Columbia for 2019. We also computed an estimate of depression prevalence among US adults with and without diabetes in 2019, stratified by age group, sex, race and ethnicity, education level, annual household income, and health insurance coverage. Weighted estimates were computed by using complex survey analytical procedures, and prevalence estimates were age-adjusted to the 2000 US Standard Population by using 4 age groups: 18–44, 45–64, 65–74, and ≥75 years. Estimates were shown only when they were statistically reliable (ie, relative standard error ≤30). Otherwise, they were suppressed. All analyses were conducted by using SAS software (version 9.4; SAS Institute Inc) and SAS-callable SUDAAN (version 11.0.1; Research Triangle Institute) to account for the complex survey sampling design.

We estimated the overall and state-specific trends in the prevalence of depression by diabetes status. We used joinpoint regression to assess trends over time (10,11). Joinpoint trend analysis can model trends over time by identifying statistically significant changes in linear trends throughout the study period. One or more time periods can be identified, in which the direction and/or magnitude of the trend changes for each time period (10,11). The annual percentage change (APC) for each identified period represents the model-estimated average annual change in prevalence. We considered trends significant if they had a 2-sided *P* value <.05.

## Results

In 2019, more than 400,000 US adults from all 50 states (except New Jersey) and the District of Columbia participated in the annual survey. Nearly 14 percent of the surveyed adults reported that they had been diagnosed with diabetes. Among those with diabetes, mean age was 61.1 years. More than half (58.4%) of those with diabetes identified as non-Hispanic White, 16.1% as non-Hispanic Black, 17.8% as Hispanic, and 7.7% as non-Hispanic other. Almost half of adults with diabetes (49.4%) had an annual household income less than $35,000, and most (91.8%) reported that they had health insurance coverage at the time of the survey. Adults without diabetes were, on average, younger and had a higher education level (Table 1).

**Table 1:** Characteristics of US Adults Aged ≥18 Years, by Self-Reported Diabetes Status, 50 States and the District of Columbia, Behavioral Risk Factor Surveillance System, 2019^a^

Characteristic	Diabetes	No diabetes
**Sample size, no.**	55,881	353,104
**Weighted sample size, no.**	27,496,213	221,532,054
**Age group, y**		
Mean age	61.1	46.0
18–44	12.2	50.2
45–64	43.2	31.3
65–74	26.7	11.0
≥75	17.8	7.5
**Sex**		
Men	50.4	48.5
Women	49.6	51.5
**Race or ethnicity**		
Hispanic	17.8	16.7
Non-Hispanic Black	16.1	11.4
Non-Hispanic White	58.4	63.1
Non-Hispanic other^b^	7.7	8.7
**Education level**		
Less than high school graduate	20.6	11.8
High school graduate or equivalent	29.9	27.5
Some college	30.4	31.1
College graduate	19.1	29.5
**Annual household income, $**		
<35,000	49.4	33.4
35,000–<50,000	13.5	12.7
50,000–<75,000	13.4	15.2
≥75,000	23.7	38.7
**Health insurance coverage**		
Insured	91.8	86.4
Uninsured	8.2	13.6

a All values are weighted percentages unless otherwise noted. All *P* values are < .001 when comparing characteristics by diabetes status. *P* values are from χ^2^ tests except for the mean age comparison, in which a 2-sample *t* test was used.

b Includes non-Hispanic American Indian and Alaska Native, non-Hispanic Asian and Pacific Islander, and multiracial groups.

**Figure 1:**
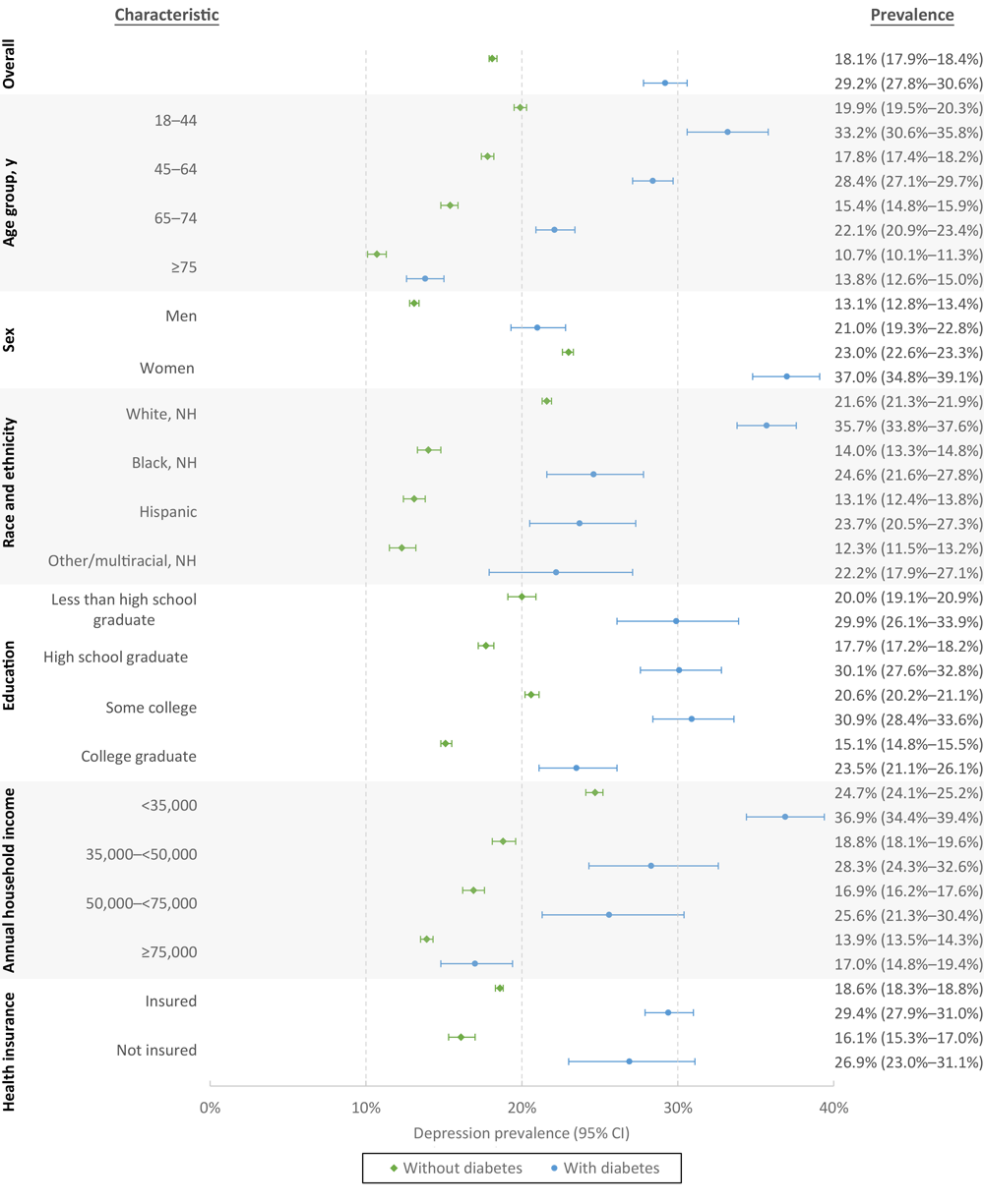
Age-adjusted prevalence of depression among US adults aged ≥18 years by diabetes status and sociodemographic characteristic, Behavioral Risk Factor Surveillance System, 2019. From 2-sample *t* tests, difference in prevalence by diabetes status is significant at *P* ≤ .01. Abbreviation: NH, non-Hispanic.

When examining depression prevalence among adults with and without diabetes, age-adjusted prevalence was significantly higher (all *P* values <.01) among those with diabetes compared with those without diabetes across all demographic and socioeconomic subgroups (Figure 1). Overall, in 2019, the age-adjusted prevalence of depression among adults with diagnosed diabetes was 29.2%, 11.1 percentage points higher than those without depression. Among adults, regardless of their self-reported diabetes status, those who were aged 18 to 44 years, women, non-Hispanic White, or with an annual household income under $35,000 were more likely to be diagnosed with depression than those in the comparison groups (Figure 1). Among adults aged 18 to 44, 33.2% of those with diabetes had prevalent depression while prevalence was 19.9% among those without depression, a difference of 13 percentage points. Among adults 75 years or older, there was a 3 percentage point higher depression prevalence among adults with diabetes compared with those without diabetes. A similar disparity was also observed among women compared with men, and among adults with the lowest annual household income compared with those reporting the highest income.

**Figure 2:**
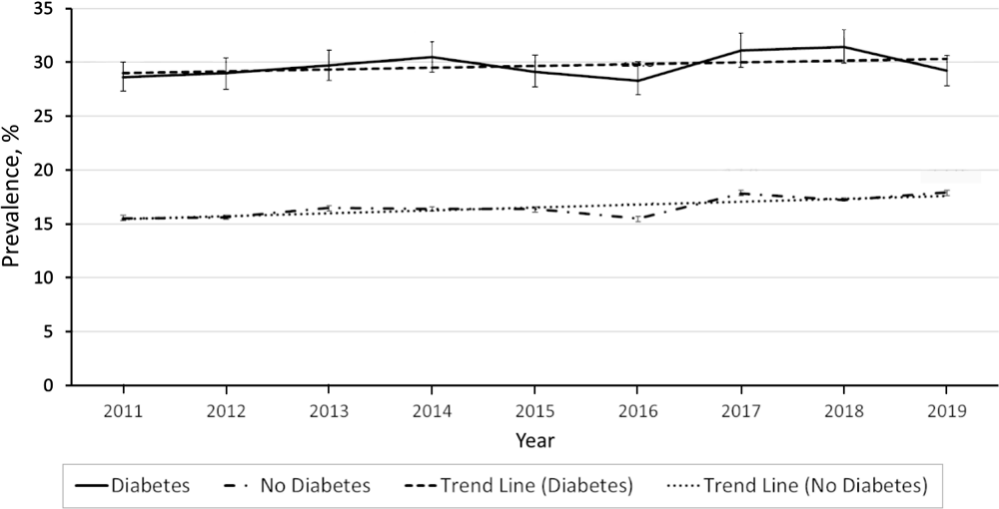
Age-adjusted depression prevalence by diabetes status, Behavioral Risk Factor Surveillance System, 2011–2019. Error bars indicate 95% CIs.

In 2019, the overall median prevalence of depression among surveyed adults with diabetes was 31.9%. State-specific depression estimates among adults with diabetes varied widely, ranging from 17.3% in California and 19.9% in Nevada to 47.0% in West Virginia and 47.9% in Kentucky. On a year-to-year basis in the overall population, depression prevalence estimates varied only slightly (Figure 2). From 2011 to 2019, the prevalence of depression was relatively stable for adults with diabetes (from 28.6% to 29.2%), though it increased slightly for those without diabetes from 15.5% to 17.9%. Over the 9-year study period, the average APC was 1.6% (95% CI, 0.4%–2.8%, *P* = .015). The prevalence of depression was consistently more than 10 percentage points higher among adults with diabetes than among those without the disease.

State-specific trends in observed depression prevalence among adults with diabetes are shown in Table 2. In 2019, although it varied by state, the depression prevalence among adults with diabetes was highest in states with a higher diabetes burden such as Kentucky (47.9%), West Virginia (47.0%), and Maine (41.5%).

**Table 2:** State-Specific Age-Adjusted Depression Prevalence Among US Adults With Diabetes, Behavioral Risk Factor Surveillance System, 2011−2019

State	2011	2013	2015	2017	2019	Time Period 1		Time Period 2	
						Years	APC (95% CI)	Years	APC (95% CI)
Alabama	38.6	31.2	37.1	42.8	29.8	2011–2019	0.9 (−3.1 to 5.1)	NA	NA
Alaska	36.5	20.0	33.7	28.2	34.8		−0.1 (−6.9 to 7.3)		
Arizona	34.9	34.0	37.3	27.0	26.1		−5.1 (−8.2 to −1.9)		
Arkansas	38.0	37.5	35.7	33.7	26.7		−1.9 (−4.8 to 1.1)		
California	16.2	19.9	19.3	22.8	17.3		3.3 (−0.9 to 7.7)		
Colorado	31.3	31.8	28.6	26.3	24.2		−3.4 (−6.3 to −0.3)		
Connecticut	27.3	32.9	31.9	30.7	22.5	2011–2014	12.1 (−7.1 to 35.2)	2014–2019	−7.1 (−14.7 to 1.2)
District of Columbia	29.4	35.6	22.6	21.6	25.4	2011–2019	−3.5 (−7.7 to 0.9)	NA	NA
Delaware	22.5	27.1	25.1	30.6	33.1		3.8 (−1.7 to 9.7)		
Florida	31.2	35.6	31.5	28.7	25.8		−4.0 (−6.2 to −1.7)		
Georgia	27.4	31.5	34.8	27.6	21.2		−1.9 (−6.4 to 2.8)		
Hawaii	20.7	17.6	15.1	17.6	21.9		1.9 (−4.7 to 8.8)		
Idaho	41.2	34.1	36.4	38.8	39.1		−0.7 (−5.4 to 4.4)		
Illinois	17.2	22.0	24.0	28.9	31.5		5.9 (2.6 to 9.3)		
Indiana	34.4	27.8	27.3	41.8	28.9		0.2 (−4.7 to 5.3)		
Iowa	28.3	36.2	28.2	37.5	29.3		−0.9 (−4.7 to 3.1)		
Kansas	27.0	31.4	35.4	38.8	33.3		3.5 (0.6 to 6.5)		
Kentucky	40.8	37.6	31.6	46.0	47.9		2.4 (−0.8 to 5.8)		
Louisiana	30.2	29.1	32.2	31.0	32.2		2.0 (−0.9 to 4.9)		
Maine	44.6	36.2	42.1	43.2	41.5		0.2 (−2.4 to 2.9)		
Maryland	21.5	24.9	26.9	32.7	24.3		0.0 (−4.5 to 4.6)		
Massachusetts	27.6	33.1	30.0	27.1	33.7		1.2 (−2.7 to 5.2)		
Michigan	35.1	35.1	29.5	35.1	34.0		1.1 (−2.1 to 4.4)		
Minnesota	29.1	41.5	33.1	32.2	30.9		−0.6 (−5.0 to 3.9)		
Mississippi	31.8	32.6	26.0	32.3	38.1		2.7 (−0.7 to 6.2)		
Missouri	42.4	39.2	42.9	37.4	33.4		−1.5 (−5.6 to 2.8)		
Montana	47.5	37.1	33.5	40.6	29.7		−2.7 (−7.2 to 2.1)		
Nebraska	29.8	30.5	32.6	34.2	26.3	2011–2017	1.8 (−0.6 to 4.2)	2017–2019	−11.6 (−23.4 to 2.1)
Nevada	27.5	32.3	30.0	25.9	19.9	2011–2019	0.2 (−5.6 to 6.4)	NA	NA
New Hampshire	42.3	33.5	33.2	32.7	36.5		−0.3 (−4.5 to 4.1)		
New Jersey	21.1	25.2	24.9	18.2	NA^a^	2011–2018	−0.6 (−6.3 to 5.5)		
New Mexico	31.8	29.8	37.6	31.0	31.7	2011–2019	−0.5 (−3.5 to 2.7)		
New York	26.4	27.9	28.6	27.2	24.0		−1.9 (−4.6 to 0.8)		
North Carolina	28.3	34.7	35.3	30.1	37.6		1.2 (−1.5 to 3.9)		
North Dakota	39.2	35.8	24.3	35.8	26.3		−1.7 (−7.7 to 4.6)		
Ohio	27.2	37.5	39.4	39.0	36.2		1.7 (−1.8 to 5.3)		
Oklahoma	36.9	46.5	40.3	32.1	33.4		−2.1 (−5.8 to 1.7)		
Oregon	45.4	37.4	37.1	37.0	38.8		0.7 (−3.9 to 5.4)		
Pennsylvania	36.2	37.5	26.8	38.0	37.6		0.1 (−3.5 to 3.9)		
Rhode Island	40.9	33.3	46.8	30.0	26.9		−4.1 (−10.9 to 3.3)		
South Carolina	20.5	30.8	31.5	32.1	32.1	2011–2013	23.4 (4.9 to 45.3)	2013–2019	0.5 (−2.3 to 3.4)
South Dakota	29.7	28.4	28.3	30.4	35.7	2011–2019	1.7 (−2.3 to 5.9)	NA	NA
Tennessee	40.1	30.3	32.7	44.7	39.3		3.9 (−0.2 to 8.2)		
Texas	28.3	23.0	24.9	30.6	28.2		1.2 (−4.4 to 7.1)		
Utah	35.0	33.1	31.1	37.1	30.7		−0.4 (−2.7 to 2.0)		
Vermont	37.3	44.1	34.6	41.5	31.7		−2.4 (−6.3 to 1.6)		
Virginia	23.9	24.2	24.4	28.8	32.0		2.3 (−1.1 to 5.9)		
Washington	37.7	35.8	36.5	37.0	34.9		−0.9 (−1.6 to −0.2)		
West Virginia	34.6	40.6	36.6	38.1	47.0		2.8 (0.0 to 5.7)		
Wisconsin	18.0	29.5	22.3	27.1	37.0		2.1 (−6.4 to 11.4)		
Wyoming	28.2	42.1	35.7	33.1	32.8		−0.3 (−6.7 to 6.4)		

Abbreviations: APC, annual percentage change; NA, not applicable.

a New Jersey had no survey data in 2019. Prevalence is shown for every other year over the study period for brevity. APC represents the estimated mean percentage change each year in depression prevalence for each period identified by joinpoint regression. APC for 1 time period is shown when joinpoint regression modeled prevalence over the study period as a monotonic linear trend. APC for 2 time periods is shown when joinpoint regression identified a change in linear trend. APC for 6 states (Arizona, Colorado, Florida, Illinois, Kansas, and Washington) was significant (*P* < .05).

**Figure 3:**
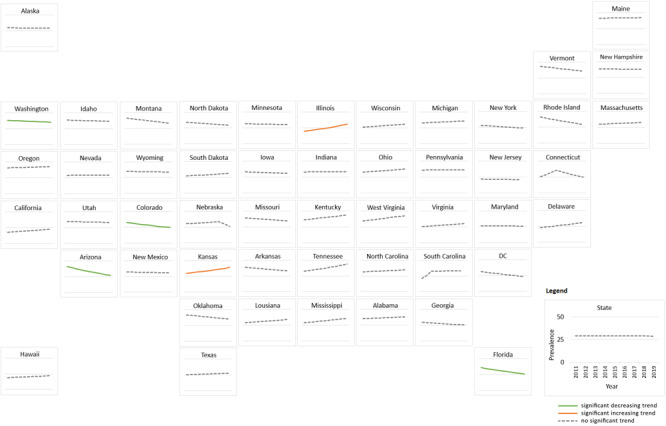
State-specific trends in annual age-adjusted depression prevalence among US adults with diabetes, 2011–2019. Trend lines for depression prevalence, as estimated by joinpoint regression, are shown for each state. Abbreviation: DC, District of Columbia.

Based on APC values, 4 states (Arizona, Florida, Colorado, and Washington) had significant decreasing trends between 2011 and 2019 (Figure 3). Their respective APC values and 95% CIs were −5.1% (−8.2% to −1.9%) in Arizona, −4.0% (−6.2% to −1.7%) in Florida, −3.4% (−6.3% to −0.3%) in Colorado, and −0.9% (−1.6% to −0.2%) in Washington. In contrast, Illinois and Kansas showed significant upward trends with APC values of 5.9% (2.6%–9.3%) and 3.5% (0.6%–6.5%), respectively. Other states showed visible increasing or decreasing trends, but these trends were not significant because of wide variability in annual prevalence estimates.

## Discussion

Throughout the study period of 2011 through 2019, the prevalence of depression was relatively stable among US adults with diagnosed diabetes (around 29%) while it increased modestly but significantly among adults without diabetes (from approximately 16% to 18%). In 2019, the overall prevalence of depression was more than 11 percentage points higher among adults with diabetes than those without diabetes, a disparity reflected in previous studies (8,9). Among adults with or without diabetes, depression prevalence was, on average, highest in the youngest age groups and among women, as previously reported (5,12). Sociodemographic and socioeconomic markers did not show a consistent association with depression prevalence.

### Demographic indicators

Adults who identified as non-Hispanic White had the highest mean prevalence of depression compared with other groups, a finding reflected in prior studies suggesting that non-Hispanic White adults have a higher prevalence of mental health conditions compared with racial and ethnic minority groups (13). However, other evidence also suggests that among racial and ethnic minority groups, disease severity, treatment rates, and outcomes of mental health conditions may be worse than in non-Hispanic White adults (13,14). Additionally, underreporting of mental health conditions such as depression may be common among racial and ethnic minority groups because of factors such as cultural norms that do not view mental well-being as a health care issue and perceived stigmatization (15,16). Efforts to improve the burden of depression and other mental health conditions may include training a more diverse health care workforce to improve cultural competency and providing culturally sensitive outreach and education (13).

In addition to differences by race and ethnicity, adults with the highest annual household incomes had the lowest mean depression prevalence compared with those with the lowest annual household incomes, likely reflecting the tendency for lower socioeconomic status to be associated with prevalent depression (17). Given that cost can be a major barrier to mental health care regardless of diabetes status (18), addressing cost barriers, such as through medical assistance programs for low-income people, can be vital to address their disproportionate burden of diabetes and depression (19).

The higher age-adjusted depression prevalence among adults with diabetes compared with those without diabetes was more pronounced in certain sociodemographic subgroups. Although depression is reported more frequently among younger adults, women, and those with lower socioeconomic status in the general population (5,17,20), the observed differences in our study suggest that diabetes may exacerbate these disparities. In these higher risk groups, diabetes distress may be more likely to occur, leading to a greater risk of depression (21). For example, the age disparity in depression prevalence in the general population may be further exacerbated by diabetes because of reduced psychologic resilience among younger adults (22) to manage stressors such as diabetes distress, which is reported to occur more frequently among younger populations (23). Similarly, among adults with diabetes, those of lower socioeconomic status may be more likely to report diabetes distress from cost-related difficulties in disease management (24). Lastly, while it is well-documented that depression is reported more frequently in women compared with men, which may be due to underreporting among men (25), it is unknown why this difference is exacerbated among adults with diabetes. Regardless, current levels of depression screening in the clinical setting may be insufficient (26) despite recommendations for the general population (27), who are likely at lower risk of depression compared with adults with diabetes. Furthermore, among adults with diabetes, particular attention to detection and screening of depression may be needed for those groups, such as younger adults, who are potentially at higher risk of depression.

### State-level variation

Although most state-level trends in depression prevalence among adults with diabetes showed relatively stable prevalence, point prevalence by state varied considerably. As the etiology of depression is complex and multifactorial, involving biologic, genetic, environmental, and social factors (28), it is difficult to empirically assess potential mechanisms for the observed geographic distribution. However, some similarities exist with the geographic distribution of other conditions such as heart disease and obesity (29,30), with much of the disease burden concentrated in the southeastern US. These similarities suggest that risk factors shared with other conditions that are more prevalent in the Southeast, such as lower socioeconomic status and a greater comorbidity burden (31), may be a potential mechanism underlying the distribution of state-level depression prevalence. In contrast, while the Southeast comprises a large proportion of rural residents compared with other regions, there is limited recent evidence supporting an association between rurality and either a higher or lower depression prevalence in the US (32). Moreover, the true geographic distribution of depression prevalence among adults with diabetes may differ from that of other conditions, as differences in methods of capturing data on prevalent diabetes can result in different geographic distributions of disease (33). Therefore, it is uncertain what factors best explain the geographic distribution of depression prevalence among adults with diabetes. Future longitudinal nationwide studies capturing detailed information across different domains (eg, lifestyle factors, laboratory measures, medical history) and regions of the US may best resolve this uncertainty.

### Comorbid mental health conditions

Among adults worldwide, a high degree of comorbidity has been reported between diabetes and depression as well as other mental health conditions. A systematic review published in 2012 reported that among adults from populations worldwide, compared with adults without diabetes, prevalence of depression was more than 3 times higher among adults with type 1 diabetes and nearly twice as high among adults with type 2 diabetes (34). A review assessing generalized anxiety disorder reported similar findings of elevated prevalent generalized anxiety disorder among adults with diabetes compared with those without diabetes (35). Similarly, a study using 2007 BRFSS survey data examined the association between diagnosed diabetes and serious psychological distress among adults and found the crude prevalence of serious psychological distress to be twice as high among adults with diabetes than among those without diabetes (9).

Several theories explain the bidirectional relationship between diabetes and depression in which each condition can present as a risk factor for the other and exacerbate the existing disease (36). Diabetes can lead to psychological stress and poor management of the disease such as unhealthy eating habits and lack of adequate physical activity, thereby increasing risk of depression (36,37). Likewise, depression itself can also lead to similar behaviors, increasing risk of diabetes. Both conditions also may share biologic risk factors, such as a heightened inflammatory state and a dysregulated hypothalamic-pituitary-adrenal axis, as well as environmental factors such as an adverse neighborhood physical environment and early life factors such as fetal malnutrition and maternal stress (37). Given the complex relationship between depression and diabetes, a need arises for effective secondary prevention of depression among adults with diabetes as well as addressing shared risk factors to prevent both conditions. When a patient is first diagnosed with diabetes, potential mental health effects may be considered, with consideration of psychiatric comorbidity as a common complication in a similar vein as other outcomes such as cardiovascular complications. Screening and maintenance of mental health may also be considered as part of routine care, in conjunction with lifestyle and/or pharmaceutical management of glycemic control.

### Strengths and limitations

The primary strength of this report is the large sample size, as the overall sample size for each annual survey was consistently over 400,000. Thus, we were able to generate statistically reliable estimates for trends in state-specific prevalence of depression by self-reported diabetes status in all US states and the District of Columbia. Our findings in this report are subject to limitations. First, BRFSS is a cross-sectional survey that gathers health and health behavior–related information by using a telephone-based interview. Self-reported information may be subject to bias or misclassification or both. In particular, respondents were asked if they were ever told by a health professional that they had depression, which may not reflect the participant’s current depression status, given that depression can be chronic or episodic. Likewise, questionnaire items measuring self-reported depression and diabetes may underestimate true prevalence because of social desirability bias or being unaware of diabetes status. Depression in particular may be underreported because of perceived stigmatization or cultural norms. Additionally, low response rates for individual states might affect the accuracy of prevalence estimates, although sampling weights can help adjust for nonresponse bias.

### Conclusions

Compared with prevalence among their counterparts without diabetes, prevalence of depression among US adults with diabetes was consistently over 10 percentage points higher. Over the last decade, the prevalence of depression in US adults with diabetes has not changed significantly, although trends varied by state. Ongoing surveillance of diabetes and depression is essential to monitor changes at the local and state level. Disease surveillance, risk factor prevention, and increased screening may lead to effective means of comprehensive disease prevention for both diabetes and depression.
